# Gut microbiota development in very preterm infants following fortification of human milk

**DOI:** 10.1128/msystems.00916-24

**Published:** 2025-02-21

**Authors:** Lin Yang, Yan Hui, Per Torp Sangild, Witold Piotr Kot, Lise Aunsholt, Gitte Zachariassen, Ping-Ping Jiang, Dennis Sandris Nielsen

**Affiliations:** 1 Section for Comparative Paediatrics and Nutrition, Department of Veterinary and Animal Sciences, University of Copenhagen, Frederiksberg, Denmark; 2 Department of Food Science, University of Copenhagen, Frederiksberg, Denmark; 3 Department of Neonatology, Rigshospitalet, Copenhagen University Hospital, Copenhagen, Denmark; 4 Department of Neonatology, Hans Christian Andersen Children’s Hospital, Odense University Hospital, Odense, Denmark; 5 Department of Clinical Research, University of Southern Denmark, Odense, Denmark; 6 Department of Plant and Environmental Sciences, University of Copenhagen, Frederiksberg, Denmark; 7 Department of Clinical Medicine, University of Copenhagen, Copenhagen, Denmark; 8 Open Patient Data Explorative Network, Odense University Hospital, Odense, Denmark; University of South Florida, Tampa, Florida, USA

**Keywords:** gut microbiota, preterm infant, nutrient fortifier, bovine colostrum, enteral feed, human milk, birth mode

## Abstract

Very preterm infants (VPIs) are born with an immature gut and predisposed to gut microbiota dysbiosis-related diseases, for example, necrotizing enterocolitis. Although fortification of human milk is required for these infants, the optimal fortifier remains uncertain. Bovine colostrum (BC), rich in protein and bioactive components, could serve as an alternative to conventional fortifiers (CF). The gut microbiota (GM) of 225 VPIs fed human milk fortified with either BC or CF (FortiColos study, NCT03537365) was profiled by 16S rRNA gene amplicon sequencing of fecal samples collected before, and after 1 and 2 weeks of fortification. Birth mode exhibited transient effects on the microbial community structure shortly after birth, with cesarean section-born VPIs dominated by *Firmicutes*, whereas vaginally born VPIs were dominated by *Proteobacteria*. This birth mode-derived difference diminished with age and disappeared around 1 month after birth. Fortifier type affected the microbial community structure to a modest extent, but no specific taxa significantly differed between the BC and CF groups. Fecal pH, increased by BC, was positively correlated with *Staphylococcus* and *Corynebacterium* and negatively with *Bifidobacterium* abundance. Change in the relative abundance of *Staphylococcus* was negatively correlated with body weight gain. Collectively, fortification of human milk with BC or CF does influence the GM of VPIs but only to a modest extent during early life. Birth mode appears to have a significant, but temporary influence on the GM during this period.

**IMPORTANCE:**

Early life is a key period for gut microbiota (GM) establishment, where enteral feeding plays a significant role. This is also the case for infants born preterm, who, due to their immature gut, are at a high risk of developing GM dysbiosis-related diseases. Human milk is the optimal feed for preterm infants, but it requires fortification to reach adequate levels of especially protein. Only a few studies have investigated the impact of fortifiers on GM development in preterm infants. Here, we demonstrate that two different bovine milk-based fortifiers, bovine colostrum and a conventional fortifier based on mature bovine milk, exhibit limited effects on the microbial community structure of very preterm infants. These findings suggest that although great care in terms of optimally maturing the preterm infant GM should be taken, the choice of fortifier only has limited impact. In clinical practice, the choice of fortifier can thus be fully focussed on optimizing preterm infant nutrition.

**CLINICAL TRIALS:**

This study is registered with ClinicalTrials.gov as NCT03537365.

## INTRODUCTION

Very preterm infants (VPIs, born before 32 weeks of gestation) have immature organs including the gut and immune systems and are highly susceptible to extrauterine growth restriction (EUGR) and gut and immune complications, such as necrotizing enterocolitis (NEC) and late-onset sepsis (LOS) (1, 2). Mother’s own milk (MOM) and donor human milk (DHM) are the preferred feed for VPIs but do not contain adequate nutrients and energy, thus requiring nutrient fortification (3). Most nutrient fortifiers are based on bovine milk (bovine milk-based fortifier [BMBF]), generally being subject to extensive processing involving protein pre-hydrolysis and often adding plant-based ingredients (4). Such fortifiers have been associated with an increased risk of gut-related complications (5). Human milk-based fortifiers (HMBFs) are not widely available, and their clinical benefits are not consistently proven (6–9). Bovine colostrum (BC), from the first few milkings of dairy cows after parturition, contains a high level of protein and many bioactive factors with antibacterial and immunomodulatory activities (10). In preterm pigs as models for preterm infants, BC increases body growth and prevents gut complications when fed exclusively or supplemented to human milk (11–15). A pilot trial (*n* = 50) showed that BC-supplemented MOM was well tolerated in VPIs (16, 17). In two subsequent, larger randomized controlled trials (RCTs), the feasibility of using BC (vs preterm formula) as a supplement to MOM in the first weeks of life (*n* = 350) (18) or as a fortifier to human milk (vs conventional fortifier [CF], *n* = 232) (19) was assessed. Both trials showed that VPIs who received BC had similar growth and clinical outcomes as their comparators (18, 19).

The gut is colonized by a myriad of microorganisms in the perinatal period. Compared with that of term-born infants, the gut microbiota (GM) of VPIs is characterized by lower species diversity (20, 21), a higher abundance of facultative anaerobes, such as *Staphylococcus*, *Enterococcus*, *Klebsiella*, *Enterobacter,* and *Escherichia,* and a lower abundance of bifidobacteria and lactobacilli (22, 23). The aberrant GM in VPIs is associated with an increased risk of aforementioned morbidities, such as NEC and LOS, and impaired growth and neurodevelopment (24–26). However, it remains unclear whether specific GM changes are directly implicated in NEC and LOS, and how this relates to nutrient maldigestion. Birth mode, gestational age (GA), hospital environment, the type of enteral nutrition (EN), and exposure to antibiotics and probiotics have all been reported to influence the GM in preterm infants (25, 27–29). Furthermore, the GM of VPIs receiving human milk (MOM and/or DHM) differs from that in infants fed preterm formula, harboring less *Escherichia* and *Clostridium* (8, 30, 31). In a pilot study, BC supplementation decreased the relative abundance of *Lactobacillaceae* and *Enterococcaceae* in VPIs, relative to DHM (32).

Compared with highly processed BMBFs, a mildly heat-treated fortifier like BC, containing intact milk proteins (casein, whey, and immunoglobulins), lipids, lactose, and many bioactive components (10, 19), may have different effects on GM development, relative to CF (32). In our recent RCT testing effect of BC vs hydrolyzed whey protein-based CF as fortifier to human milk (FortiColos), both fortifiers improved growth to the desired level, and morbidities were similar (19). In this study, we aim to investigate whether fortification with BC or CF affects the GM in the weeks immediately after the start of fortification when the concern for gut complications, such as feeding intolerance and NEC, is particularly high.

## MATERIALS AND METHODS

### The trial and collection of fecal samples

**Fig 1:**
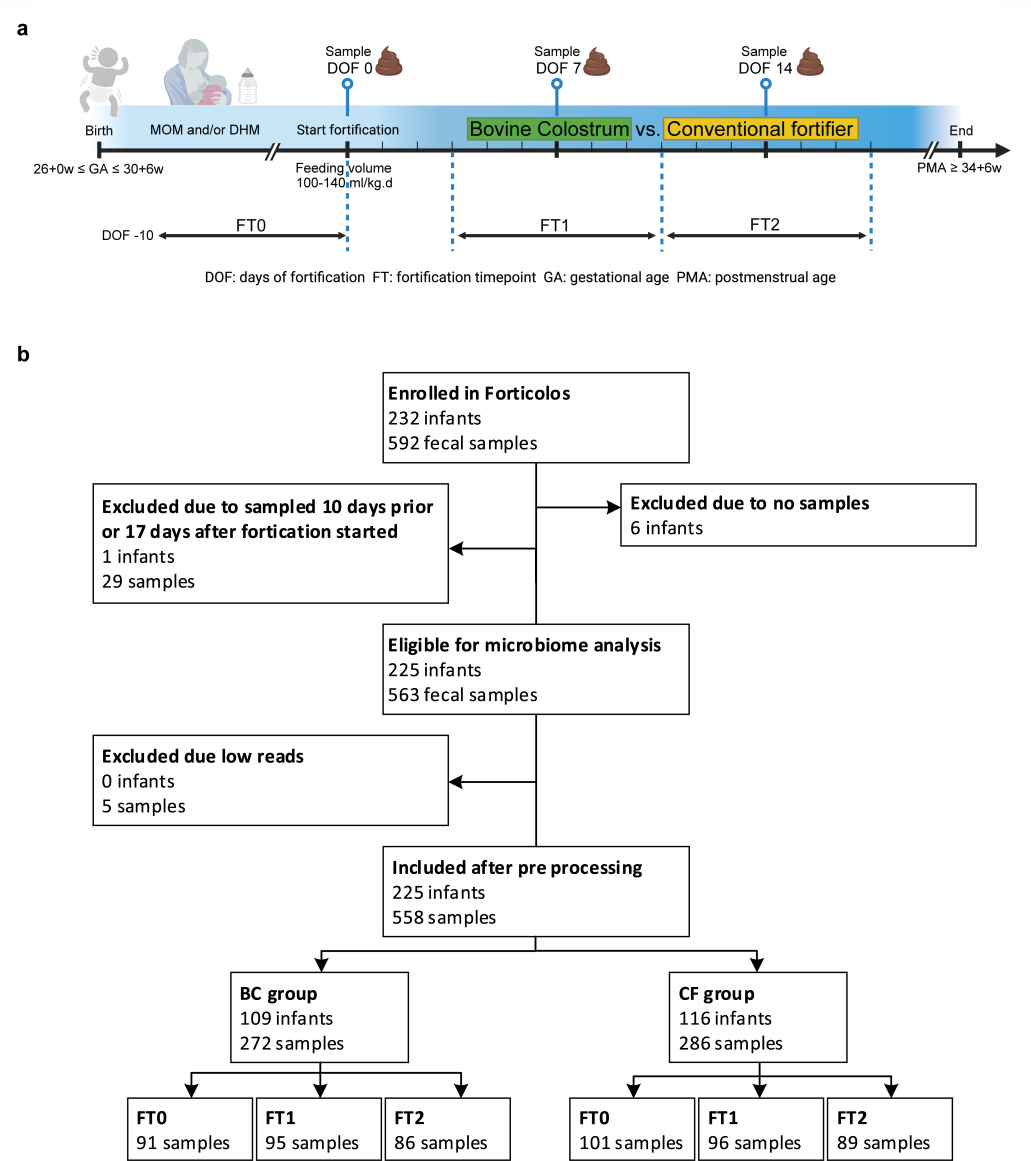
Design of the FortiColos trial and sample inclusion for the gut microbiota analysis. (**a**) Study design and fecal sample collection. Fecal samples before fortification (from −10 to 0 days of fortification, DOF, FT0), around 1 week (7 ± 3 DOF, FT1), and 2 weeks of fortification (14 ± 3 DOF, FT3) were included. Created with BioRender.com. (**b**) Infants (samples) included in the microbiota analysis. Twenty-nine and five samples were excluded due to unmatched collection time and/or low sequencing reads.

VPIs included in this study were enrolled in the FortiColos trial (Bovine colostrum as a fortifier added to human milk for preterm infants, clinicaltrials.gov registration NCT03537365). The FortiColos trial is a multicentre RCT conducted at eight hospitals in Denmark testing the feasibility of using BC as a nutrient fortifier to MOM and/or DHM for VPIs (19). These VPIs were of GA between 26 + 0 and 30 + 6 weeks and required nutrient fortification. EN was provided following the local guidelines prior to fortification. When MOM was not available or insufficient, banked DHM was used. Fortification started when EN volume reached 100–140 mL/kg/d and blood urea nitrogen (BUN) <5 mmol/L. When BUN was ≥5 mmol/L, fortification was postponed or paused until BUN <5 mmol/L. BC (BC group, ColoDan powder, Biofiber-Damino, Gesten, Denmark) was compared with a conventional BMBF (CF group, PreNan FM85, Nestlé, Vevey, Switzerland). Initially, 1.0 g of fortifier was added to 100 mL of human milk and gradually increased to a maximum of 2.8 g BC/100 mL and 4.0 g CF/100 mL, both equivalent to the maximum of 1.4 g protein/100 mL (Fig. 1a) (19, 33). Fortification continued until the infants reached postmenstrual age (PMA) 34 + 6 weeks, were early discharged, were moved to a non-participating unit, or had diseases. Probiotics (Bifiform, Ferrosan, Denmark), containing freeze-dried *Lactobacillus rhamnosus* GG (LGG; 2 × 10^9^ CFU) and *Bifidobacterium lactis* BB-12 (2 × 10^8^ CFU), were routinely used in four participating hospitals according to the local guidelines (Table S1).

**Fig 2:**
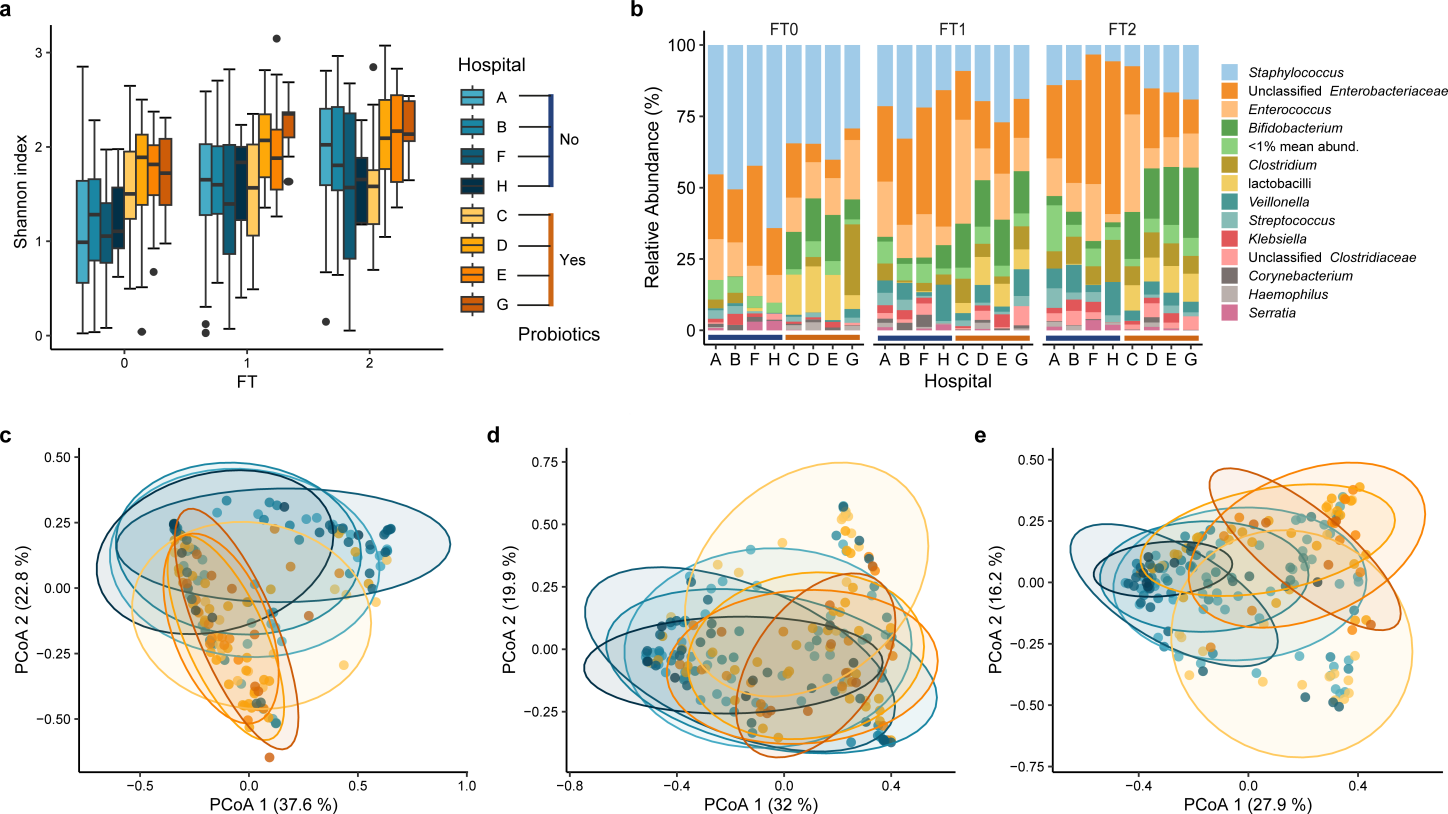
The administration of probiotics at some hospitals (C, D, E, G) intricately influence the gut microbiota relative to hospitals (A, B, F, H) not administrating probiotics. (**a**) Species diversity was assessed by the Shannon index in different hospitals. (**b**) The relative abundance of different genera according to hospital groups. Taxa that could not be classified as a specific genus were labeled “unclassified” with their upper-level taxa. Genera with mean relative abundance lower than 1% at each FT were summed up and labeled as “<1% mean abund.” (**c–e**) PCoA plot representing microbial community structure based on weighted Unifrac distance of different hospital groups. The ellipses represented 95% confidence intervals.

Characteristics of enrolled infants include demographics such as GA, gender, multiple births, small for gestational age (SGA), birth mode, day of life (DOL), hospital, and medical treatment such as basic feed, use of antibiotics, and use of probiotics until the end of intervention were prospectively collected from electronic medical records. Stool samples were collected from infant diapers at three time points, including before (fortification time-point 0 [FT0]), and 1 (FT1) and 2 weeks (FT2) after the start of the fortification (Fig. 1a; Fig. S1a). FT0, FT1, and FT2 were roughly equivalent to 2–10, 10–20, and 20–30 DOL (Fig. S1b). The samples were stored at −50 to −80°C or placed in the refrigerator (+4°C) for a maximum of 24 h before being transferred to a −50 or −80°C freezer.

### Fecal pH measurement

Fecal pH was measured in 1:1 (200 mg:200 µL sterile MilliQ water) diluted samples at room temperature by pH sensor (InLab Micro Pro-ISM equipped with SevenCompact pH meter S220, Mettler Toledo, Columbus, USA).

### GM analysis

The fecal GM was profiled by 16S ribosomal RNA (rRNA) gene V3 hypervariable region amplicon sequencing. DNA from approximately 200 mg of each sample was extracted by DNeasy PowerSoil Pro Kit (QIAGEN, Hilden, Germany) according to the manufacturer’s instructions. Library preparation followed the published protocol (34). The V3 region (forward primer NXT338: 5′-CCTACGGGWGGCAGCAG-3′, reverse primer NXT518: 5′-ATTACCGCGGCTGCTGG-3′) was used for the two-step PCR amplification. The PCR products were purified with Agencourt AMPure XP Beads (Beckman Coulter Genomics, MA, USA) and quantified with Qubit 1× dsDNA HS assay kit (Invitrogen, Thermo Fisher Scientific). PCR products were pooled in even concentrations and were pair-ended sequenced (2 × 150 bp) by NextSeq (Illumina, San Diego, CA, USA). Sterile DNA-free water was used as negative control and mock community DNA was used as positive control during DNA extraction and library preparation. Both negative and positive controls were included for sequencing.

The bioinformatics process of the sequencing data adhered to established procedures previously described (35). Specifically, the initial steps involved demultiplexing, merging, and trimming of the raw sequencing data, followed by the removal of chimera and generation of zero-radius Operational Taxonomic Units (zOTUs) using the UNOISE3 (36) algorithm implemented in Vsearch (version 2.21.1) (37). The Greengenes (13.8) (38) 16S rRNA gene database was employed as a reference database for taxonomic annotation.

### Data analysis

Characteristics of included infants including continuous and categorical variables were described by mean (standard deviation) and count (percentage), respectively. Group comparisons were conducted using one-way analysis of variance (ANOVA) for continuous variables and chi-square (χ^2^) test for categorical variables.

Analysis and visualization of the microbiota data were performed by R packages Phyloseq (39), Vegan (40), and ggplot2 (41). Raw zOTUs were rarefied at an even depth of 10,000 counts per sample. The samples with insufficient sequencing depth were excluded from the analysis (five samples; Fig. 1b). Effect of variables, including fortifiers, GA, SGA, birth mode, DOL, hospital, and use of antibiotics, on the GM was tested using distance-based redundancy analysis (dbRDA). Data on the administration of probiotics are confounded by the factor “hospital” with four hospitals using probiotics and four not (Table S1) and thus were not included in the analysis.

The species diversity (alpha-diversity), measured by the number of observed zOTUs and Shannon diversity index, was compared between the birth mode groups (cesarean section [CS] vs. vaginal birth [VB]) by linear regression with adjustment for confounders, including fortifier, GA, SGA, DOL, hospital, and use of antibiotics. The microbial community structure (beta-diversity) was assessed based on weighted UniFrac distance dissimilarity metrics and shown with principal coordinates analysis (PCoA) score plots. The difference between CS and VB infants was tested by permutational multivariate analysis of variance (PERMANOVA). Three GM types were determined in all samples according to the dominating phylum with relative abundance over 50% as *Firmicutes*-dominated, *Proteobacteria*-dominated, and another mixed pattern. Each GM type of the CS and VB infants at the same FT was compared by χ^2^ test. The comparison of infants with *Firmicutes*-dominated GM type or not between different FTs was conducted using logistic regression with adjustments for confounders within each birth mode group. DESeq2 with the same covariates was used to locate OTUs with differences in relative abundance between the two groups (42) (only tested if relative abundance was over 1% and present in over 50% of samples in either fortifier group at each FT). False discovery rate (FDR) approach was used to correct *P* values from multiple tests at each FT. Comparisons between the fortifier groups (BC vs. CF) on the species diversity based on the Shannon diversity index and the number of observed zOTUs, the microbial community structure based on weighted UniFrac, unweighted UniFrac, Bray-Curtis and Jaccard (dis)similarity metrics, and OTUs were conducted by the same methods mentioned above with adjustment for different confounders, including birth mode, GA, SGA, DOL, hospital, and use of antibiotics. A parameter, the 3-day proportion of MOM, was included in the analyses mentioned above to adjust for the effect of base feed on the GM. It was defined as the number of meals of exclusive MOM over that of all meals in the 3 days before a FT with a meal with both MOM and DHM registered as 50% MOM.

A sub-analysis was conducted to examine differences in the GM between infants born to the same mother (multiple births) and those born to different mothers (i.e., non-related infants), using Bray-Curtis dissimilarity distances. The dissimilarity values between the two groups were compared using the Wilcoxon test.

Changes in the relative abundance of the three most abundant bacterial genera (*Staphylococcus, Enterococcus* and unclassified *Enterobacteriaceae,* log10-transformed) and the species diversity (the Shannon index and the number of zOTUs) were correlated with anthropometric parameters (z-scores of body weight, body length, and head circumference) between different FTs across the fortifier groups. Body weight, body length, and head circumference were calculated into z-scores with reference to the Swedish growth charts for preterm infants (43). The value applied for the correlation was assessed by the delta of anthropometric parameters and species diversity, or ratios of the relative abundance between two FTs, respectively. Pearson correlations were adopted after outliers of ratio being removed according to three-sigma limits, and FDR approach was used to correct *P* values within the same period. The relative abundance of the 15 most abundant genera (central log-ratio transformed) was correlated with the fecal pH using Pearson correlation (44). FDR approach was adopted to correct *P* values from the correlation analyses within each analysis.

All statistical analyses were performed in R (version 4.2.1) (45). FDR-corrected *P* values were shown as *q* values. The *P* or *q* < 0.05 was regarded as statistically significant.

## RESULTS

The FortiColos trial aimed to test the feasibility of using BC as fortifier for human milk. VPIs, who were fed MOM and/or DHM, were enrolled and randomized to receive BC or CF as fortifier added to human milk. Of the 232 VPIs enrolled in the FortiColos trial, fecal samples from 225 (BC, *n* = 109; CF, *n* = 116) were collected and applied to sequencing. Samples with acceptable data quality were included in the analysis (192, 191, and 175 at FT0, FT1, and FT2, respectively; Fig. 1b). The mean and median sequencing depth of all samples were 91,251 and 82,865 reads, respectively. In total, 2,535 unique zOTUs were identified in all samples with 2,227 in the BC group and 2,351 in the CF group, respectively.

### Characteristics of the included infants

Characteristics of the 225 infants included in this study are shown in Table 1. No significant difference was found in GA, BW, sex (male or female), SGA (yes or no), multiple births (singleton or not), birth mode (CS or VB), or 3-day MOM proportion between the infants receiving BC or CF (all *P* > 0.05; Table 1). No significant difference was found between the BC and CF groups in the numbers of infants receiving probiotics at the hospitals with routine probiotics use before fortification or in the numbers of infants receiving antibiotics at any FT (all *P* > 0.05; Table 1). The proportion of infants born by CS was not significantly different between the two intervention groups (*P* > 0.05). No clear phenotypic difference was observed between infants born by CS or VB, except that the prevalence of SGA was higher in the CS infants (*P* < 0.05; Table S2).

**TABLE 1:** Characteristics of infants included for gut microbiota analysis

Characteristics	BC	CF	*P*
*N*	109	116	
GA, weeks (mean (SD))	28.7 (1.5)	28.6 (1.5)	0.51
Gender, male/female (%)	67 (62.0)/41 (38.0)	63 (54.3)/53(45.7)	0.28
Birth weight, g [mean (SD)]	1,181.5 (330.9)	1,162.1 (323.5)	0.66
SGA,^*a*^ yes/no (%)	24 (22.0)/85 (78.0)	26 (22.4)/90 (77.6)	1.00
Multiple birth, yes/no (%)	30 (27.5)/79 (72.5)	39 (33.6)/77 (66.4)	0.40
Birth mode, VB/CS (%)	35 (32.1)/74 (67.9)	29 (25.0)/87 (75.0)	0.30
Probiotic use,^*b*^ yes/no (%)	35 (32.1)/74 (67.9)	29 (25.0)/87 (75.9)	0.30
DOL, days (median [IQR])			
FT 0	7.0 [6.0, 8.5]	7.0 [6.0, 9.0]	0.35
FT 1	15.0 [14.0, 17.0]	16.0 [14.0, 18.3]	0.08
FT 2	22.0 [20.0, 24.0]	22.0 [20.0, 25.0]	0.28
3-day MOM proportion, % [mean (SD)]^*c*^			
FT 1	83.0 (29.7)	88.5 (23.5)	0.17
FT 2	84.3 (30.1)	88.0 (27.4)	0.41
Antibiotic use,^*d*^ yes/no (%)			
FT 0	41 (45.1)/50 (54.9)	36 (35.6)/65 (64.4)	0.24
FT 1	20 (21.1)/75 (78.9)	18 (18.8)/78 (81.2)	0.83
FT 2	15 (17.4)/71 (82.6)	9 (10.1)/80 (89.9)	0.23

^*a*^ SGA is defined as a birth weight (BW) Z score ≤ −2 standard deviations for GA.

^*b*^ Probiotic use is defined as using probiotics before intervention started.

^*c*^ Feeding data were recorded after fortification start.

^*d*^ Antibiotic use is defined as using antibiotics within 5 days before stool sample collation.

### Birth mode affects the gut microbiota composition in VPIs

Before assessing the effect of fortifier on VPI GM development, we investigated if other variables including fortifiers, GA, SGA, birth mode, DOL, hospital, and use of antibiotics significantly influenced GM. “Hospital” was found to significantly influence VPI GM (Table S3). However, as probiotic use is fully confounded by “hospital,” whether this difference is driven by the use of probiotics, other hospital-specific factors, or both is not possible to determine (Fig. 2; Table S1). Among all investigated variables, birth mode had the most pronounced influence on GM composition (Table S2), prompting a more in-depth investigation.

**Fig 3:**
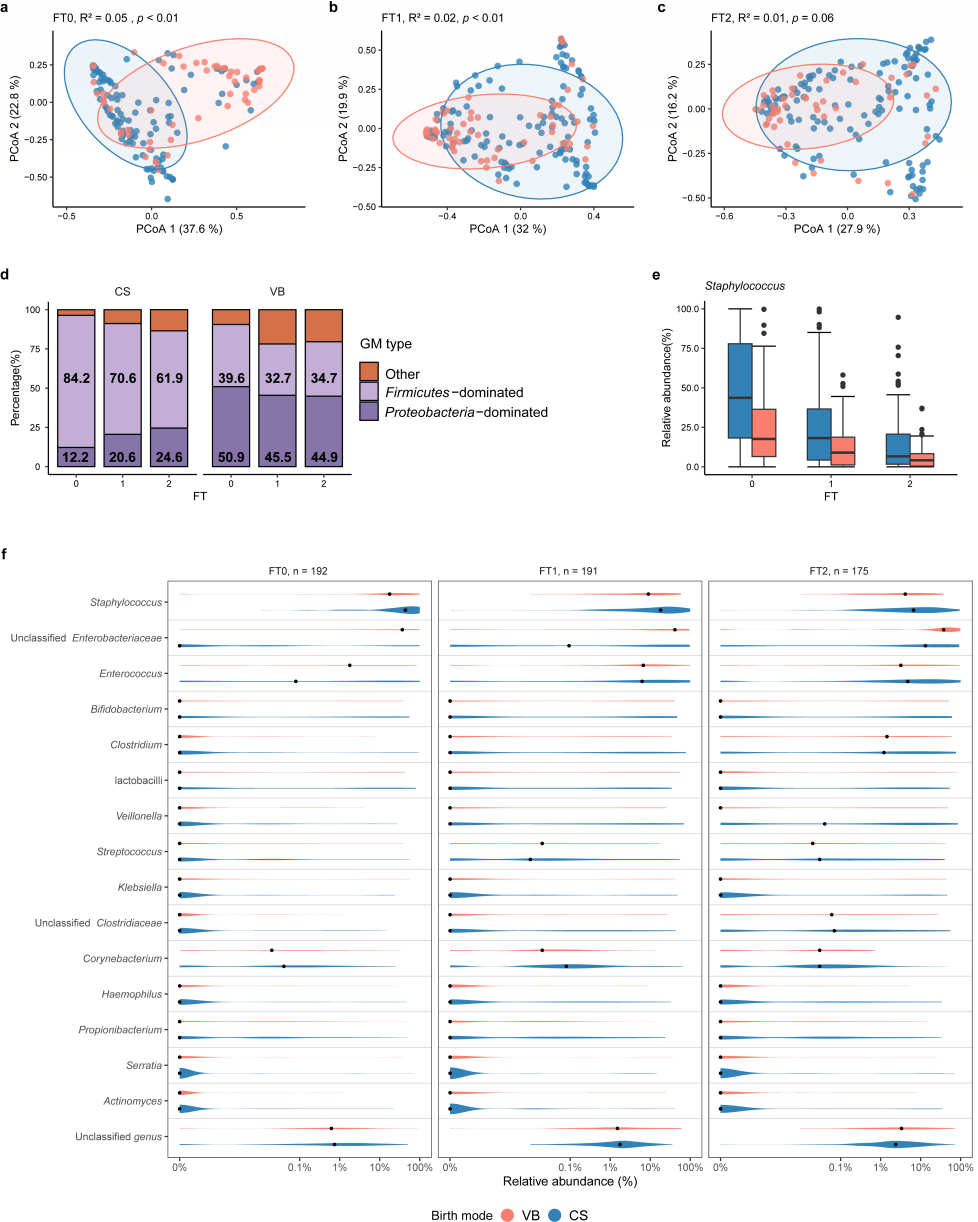
Birth mode transiently affects the gut microbiota. (**a–c**) The microbial community structure was different in VPIs born by cesarean section (CS) or vaginal birth (VB), but the difference diminishes over time. PCoA plots were based on weighted Unifrac dissimilarity metrics. The ellipses represented 95% confidence intervals. *R*^2^ and *P* values were calculated by PERMANOVA (999 permutations) and adjusted for confounders, including GA, SGA, DOL, fortification, hospital, and use of antibiotics. (**d**) CS infants were most *Firmicutes*-dominated, whereas VB infants were most *Proteobacteria*-dominated. The GM type of the CS infants changed over time. The GM type of the CS and VB infants at the same FT was compared by χ^2^ test. The comparison of infants being *Firmicutes*-dominated or not between different FTs was conducted using logistic regression with adjustments for confounders within each birth mode group. (**e**) Relative abundance of *Staphylococcus* was different in the CS and VB infants. The relative abundance of *Staphylococcus* was higher in the CS group at FT1 and FT2, as revealed by DESeq2 with adjustment of confounders before FDR correction at each FT. (**f**) Relative abundance of the most abundant 15 genera in the CS and VB infants at three FTs. The order of genera was based on the mean abundance. Median values are shown as black dots. A pseudocount of 1 × 10^−6^ was added to all relative abundance values to facilitate log-scale transform.

Of the VPIs receiving BC, 35 were VB, and 74 were born by CS, whereas among the CF fed VPIs, 29 were VB and 87 were born by CS. Birth mode (CS or VB) did not affect the species diversity as determined by the Shannon diversity index or the number of observed zOTUs at any FT (all *P* > 0.05; Fig. S2a and b). However, the microbial community structure differed between the CS and VB infants in the first 20 days postpartum (FT0, *R*^2^ = 0.05; FT1, *R*^2^ = 0.02, respectively, both *P* = 0.001; Fig. 3a and b), but the effect was smaller (*R*^2^ = 0.01) and not significant (*P* = 0.06; Fig. 3c) at FT2, indicating a decreasing influence of birth mode on GM as the VPI gets older over the first weeks.

Most CS infants had a *Firmicutes*-dominated GM (with 84.2% of infants being *Firmicutes*-dominated at FT0 and 61.9% at FT2), whereas most VB infants had a GM dominated by *Proteobacteria* (44.9%–50.9%) at all FTs (all *P* < 0.01; Fig. 3d). The number of *Firmicutes*-dominated infants decreased with time in the CS infants (FT0 vs FT1, FT0 vs FT2, both *P* < 0.05; Fig. 3d), but not in the VB infants (FT0 vs FT1 vs FT2, all *P* > 0.05; Fig. 3d). At the genus level, *Staphylococcus, Enterococcus,* and unclassified *Enterobacteriaceae* were the three most abundant genera at all three FTs (Fig. 3f). The relative abundance of *Staphylococcus* was significantly higher in the CS infants than that in the VB infants at FT1 and FT2, before (both *P* < 0.05), but not after (*q* > 0.05, DESeq2; Fig. 3e) FDR correction. Notably, the relative abundance of *Bacteroidetes* was above 2% in VB infants, whereas in CS infants, this phylum was either not detected or very low abundant (<0.01%) at any FT (Fig. S2c).

### Choice of fortifier has a limited effect on GM development during the first month of life in very preterm infants

**Fig 4:**
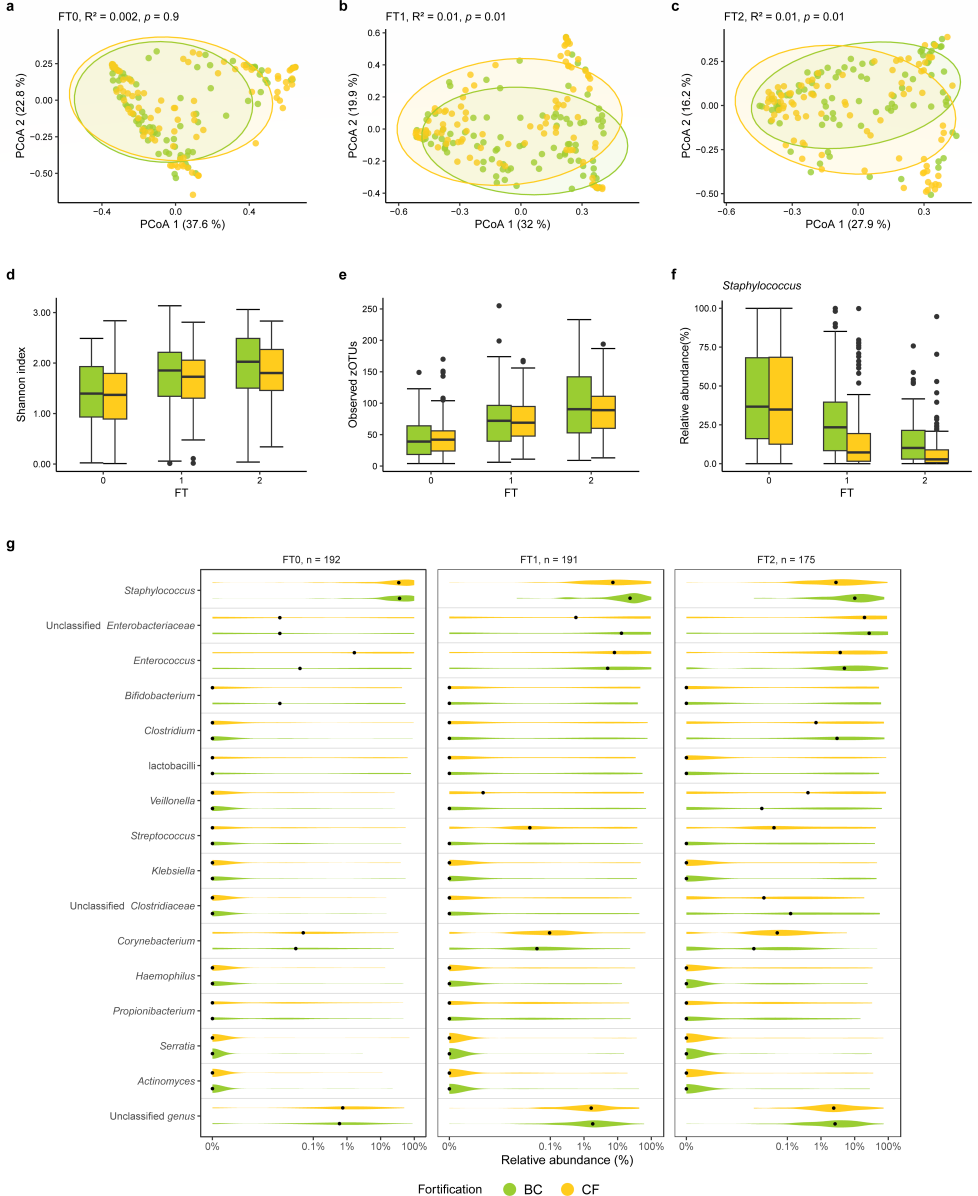
BC or CF as a human milk fortifier has limited effects on the gut microbiota of VPIs. (**a–c**) The microbial community structure differs between infants receiving BC and CF fortification. A significant difference between the fortifier groups was found at FT1 and FT2, but the variance explained by fortifiers was small. PCoA was based on the weighted Unifrac dissimilarity metrics. *R*^2^ and *P* values were calculated by PERMANOVA (999 permutations) and adjusted for confounders including GA, SGA, birth mode, DOL, hospital, and use of antibiotics. The ellipses in the plots represented 95% CI. (**d and e**) The species diversities were based on the Shannon index and the number of observed zOTUs. No significant difference was found between the BC and CF groups at each FT when compared using linear regression adjusted for the confounders. (**f**) Relative abundance of *Staphylococcus* in the BC and CF groups. The significant difference in relative abundance as revealed by DESeq2 with adjustment for confounders, disappeared after FDR correction. (**g**) Relative abundance of the top 15 abundant genera in the BC and CF groups. The order of genera was based on the mean abundance. Median values are shown as black dots. A pseudocount of 1 × 10^−6^ was added to all relative abundance values to facilitate log-scale transformation.

No significant difference was found between the BC and CF groups at any FT with respect to the Shannon diversity index or the number of observed zOTUs (all *P* > 0.05; Fig. 4d and e). However, the gut microbial community structure differed between the BC and CF groups at FT1 and FT2 (both *P* = 0.01; Fig. 4b and c) as determined by Weighted UniFrac distance metrics, but the choice of fortifier only explained a relatively small proportion of the variance (*R*^2^ = 0.01 at both FTs; Fig. 4b and c). Similar results were found when other (dis)similarity metrics, such as unweighted UniFrac, Bray-Curtis, and Binary Jaccard, were used (Fig. S3). In a subgroup analysis by birth mode, the fortifier-driven difference in the microbial community structure was only observed in the infants born by CS (*P* < 0.05; Fig. S4), but the variance explained was still modest (*R*^2^ = 0.02). A sub-analysis showed that the Bray-Curtis dissimilarity distances between infants born to different mothers (i.e., non-related infants) were significantly greater than that between infants born to the same mother, that is, multiple births such as twins (all *P* < 0.05; Fig. S5).

The relative abundance of the 15 most abundant genera in the GM can be seen in Fig. 4g. The relative abundance of *Staphylococcus* was significantly higher in the BC infants, relative to the CF infants, at FT1 (*P* < 0.05) and FT2 (*P* = 0.05, Deseq2), before but not after FDR correction (*q* > 0.05, FT1 and FT2; Fig. 4f).

To assess the influence of base feed (MOM and DHM) on the effect of fortifier (BC or CF) on the GM, 3-day proportion of MOM (*n* = 202) was included in the analyses as a confounding factor. Fortifier (BC vs. CF) was still found to affect the microbial community structure at FT1 and FT2, with the GM variation explained by fortifier and by the MOM proportion similar (all *R*^2^ ≈ 0.01; Table S4).

### Relative abundance of *Staphylococcus* correlates with body weight gain

**Fig 5:**
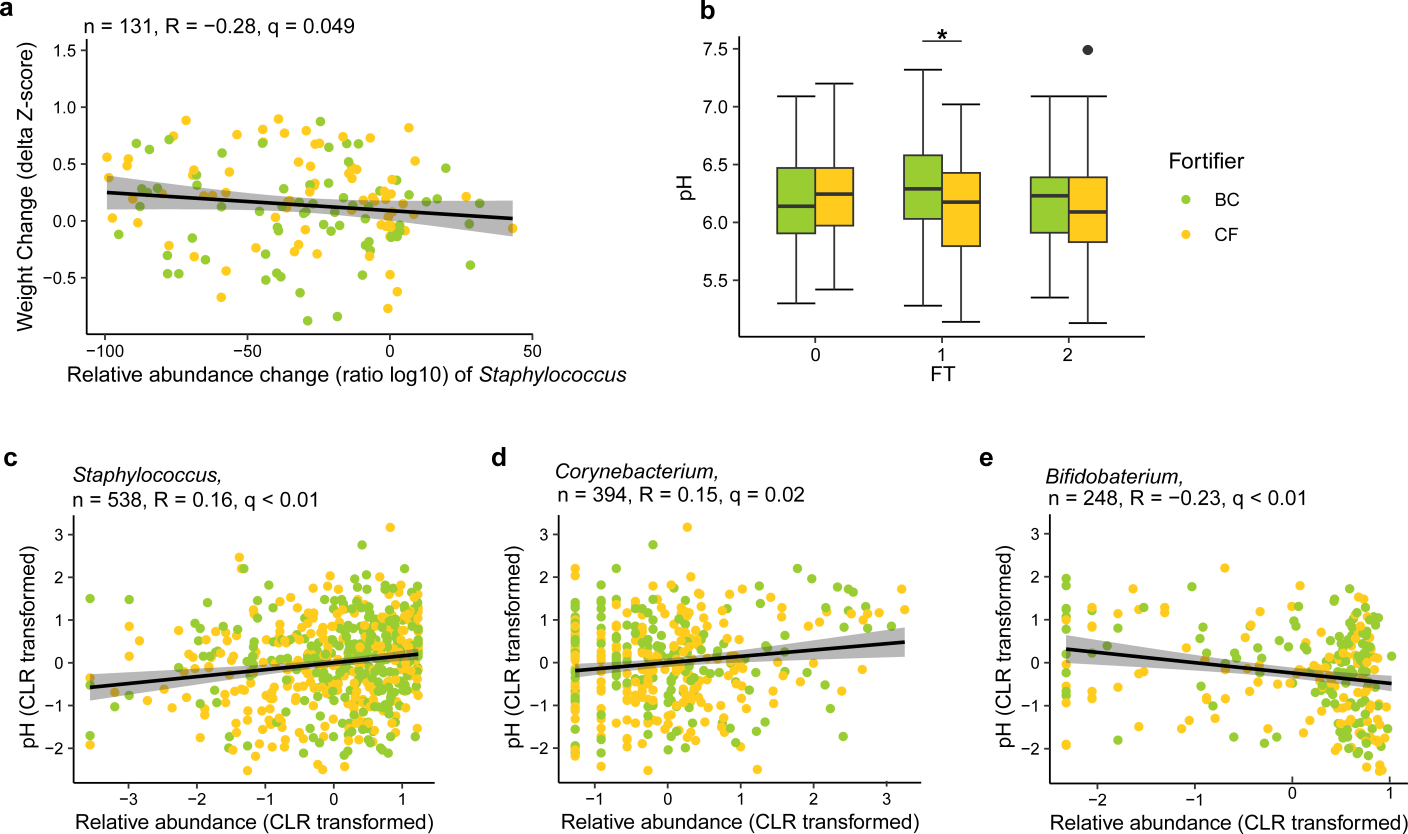
The gut microbiota is correlated with weight gain and fecal pH. (**a**) The change of *Staphylococcus* relative abundance (as determined by the ratio of log10-transformed relative abundance) was negatively correlated with body weight gain (delta body weight Z-scores) from FT0 to FT2 across the fortifier groups after FDR correction. Only 131 infants with samples and body weight data available at all FTs and within three standard deviations (SDs) were included. (**b**) Fecal pH was significantly higher in the BC group than in the CF group at FT1 (linear regression). *, *P* < 0.05. (**c**) Relative abundance of *Staphylococcus* was positively correlated with fecal pH across FTs (*q* < 0.01, *R* = 0.2, *n* = 538). (**d**) Relative abundance of *Corynebacterium* was positively correlated with fecal pH across FTs (*q* = 0.02, *R* = 0.2, *n* = 394). (**e**) Relative abundance of *Bifidobacterium* was negatively correlated with fecal pH across three FTs (*q* < 0.01, *R* = −0.2, *n* = 248). Relative abundance of the 15 most abundant genera (central log-ratio transformed) and the pH of fecal samples were correlated by Pearson correlation. *P* values of all correlations were further adjusted by FDR correction to generate *q* values.

At FT0, *Staphylococcus* dominated the GM of almost all enrolled VPIs. At FT1 and FT2, *Staphylococcus* was still among the dominant taxa but showing a decreasing trend in relative abundance (Fig. 4g). Notably, the change of *Staphylococcus* relative abundance, shown as the ratio of log10-transformed relative abundance, was negatively correlated with body weight gain (delta body weight Z-scores) from FT0 to FT2 in all infants (*n* = 131, *q* = 0.049, *R* = −0.28, Pearson correlation; Fig. 5a), indicating that weight gain was higher in the VPIs where *Staphylococcus* relative abundance decreased the most from FT0 to FT2. It was also tested if similar correlations were seen between weight gain the two other dominating taxa, *Enterococcus* and unclassified *Enterobacteriaceae*, but neither was associated with changes in body weight, body length, or head circumference (all *q* > 0.05, Pearson correlation).

### Choice of fortifier influenced fecal pH

Fecal pH was higher in the BC group than in the CF group at FT1 (6.29 ± 0.40 vs 6.14 ± 0.45, mean ± SD, BC vs CF, *P* = 0.02; Fig. 5b). The same trend was seen at FT2, although it was not significant. The relative abundance of *Staphylococcus* and *Corynebacterium* was positively correlated with fecal pH (*q* < 0.01, *R* = 0.16; *q* = 0.01, *R* = 0.15, respectively, Pearson correlation; Fig. 5c and d), meaning that the higher the fecal pH, the higher the relative abundance of these two genera. The relative abundance of *Bifidobacterium* was, on the other hand, negatively correlated with fecal pH across fortifier groups and FTs (*q* < 0.01, *R* = −0.23, Pearson correlation; Fig. 5e).

## DISCUSSION

The first weeks after the start of fortification of human milk is a critical time to secure adequate growth of VPIs without overloading the immature gut with proteins, which could otherwise predispose to intestinal complications, such as feeding intolerance and NEC (46). Such complications have been speculated to involve adverse fortification-induced changes to the GM (7, 47). Here, we investigated if fortification with two different fortifiers (BC with intact proteins and immunoglobulins, CF with hydrolyzed whey protein) influenced short-term GM development, and we coupled these data with our previous reports on clinical variables and growth outcomes of the same VPIs (19). The choice of fortifier affected GM composition, but the effects were modest and not associated with the abundance of specific GM genera. In contrast, birth mode (CS or VB) had a more pronounced impact, particularly soon after birth, and the differences between the two fortification groups were only present in CS infants.

Birth mode is known to affect the GM in term infants (48, 49), whereas the effects in VPIs are more variable (27). In this study, birth mode was found to clearly affect the GM of VPIs shortly after birth, but the effect was transient and no longer clear at 3–4 weeks of age (Fig. 3a through c). The CS VPIs harbored a GM dominated by gram-positive *Firmicutes*, whereas a larger fraction of the VB infants harbored a GM dominated by gram-negative *Proteobacteria* (Fig. 3d). This was also reflected in a tendency for the relative abundance of *Staphylococcus* being higher in CS versus VB VPIs (Fig. 3e). It is speculated that the GM in CS VPIs is more dominated by the microbes of hospital environment and maternal skin origin, such as *Staphylococcus* (21, 50). The bacteria more frequently found in the GM of term CS infants have been associated with morbidities later in life, such as asthma, infectious and inflammatory diseases, as well as obesity, suggesting that the possible involvement of the GM (51). However, whether these associations are also evident in preterm infants is uncertain, and well-designed studies testing long-term effects are required. Importantly, our findings suggest that the GM of VPIs born by CS might reconstruct to resemble that of VB infants within the first month of life, potentially alleviating the influence of an aberrant GM after CS on the later disease susceptibility of these infants.

Previous results on the GM effects of fortification in VPIs are conflicting. Two recent studies comparing GM after supplementation with fortifiers based on human or bovine milk reported significant GM effects (7, 47), whereas another study showed no effect (52). It is important, however, to evaluate not only the effects but also their magnitude. Although a significant difference was found in the effect of BC vs CF on the GM in our study, the effect was modest. The variance explained by fortifier type accounted for only ~1% of the total variance of the microbial community structure, and the effect of other factors, such as GA and antibiotics use, is comparable with that of fortifier type (Table S3). Additionally, CS infants seem more likely to respond to different fortifiers, relative to VB infants, with a significant effect of fortifier type observed only in CS infants. We speculate that the GM of VB-born infants may be more stable and resilient to perturbation than that of CS-born infants. Our results indicated that the number of *Firmicutes*-dominated infants decreased over time in the CS group but remained stable in the VB group, reflecting the higher stability of the GM in VB-born infants. This idea is further supported by a previous study showing higher GM stability of VB-born infants relative to CS-born infants up to 2 months of age (53). Of note, the VB-born infants in the mentioned study also had a longer duration of breastfeeding compared with the CS-born infants (53), which also influences the GM.

It has been suggested that human milk (MOM and DHM) as base feed has more pronounced effects on shaping the GM in VPIs than the milk fortifiers used (52). Inclusion of the 3-day proportion of MOM (as a measure of base feed composition) in the statistical models did not change the results. The variance in the GM community structure explained by fortifier type and the base feed composition was similar (*R*^2^ ≈ 0.01), suggesting the comparable influence of fortifiers and base feed on the GM composition at least in the period of our trial. Marked effects of base diet on GM may develop later, consistent with the longer period of observation in the earlier study (DOL 32–60) (52). Like for fortifiers, GM differences related to base feed may be explained by both the amount and composition of proteins in the feed. In our study, the VPIs on BC received ~10% more dietary protein than CF infants (19), and it remains unknown if the differences in GM composition between the groups were due to higher protein load or BC-related differences in feed protein composition (54, 55). In our study, protein in fortifiers accounted for 21-22% of the daily protein intake at FT1, and this proportion increased to 26% at FT2 when fortification amount reached a plateau 3–4 weeks after birth. Our study suggests that differences in both the human milk base feed and choice of fortifier exert only modest effects on GM composition in the first month of life, the time when the risk of GM-related gut complications is the highest.

Several studies have shown that mother and offspring often share microbial taxa and strains, highlighting the potential for maternal-infant transmission (56, 57). In line with this, we also observed that the GM of infants from multiple births (e.g., twins and triplets) exhibited greater similarity compared with those from single births. Importantly, maternal factors during pregnancy, such as antibiotic use, probiotics, and diet, can alter the maternal microbiota, which may subsequently influence the infants’ GM (58). However, in our cohort, the lack of maternal samples and data on maternal exposures (e.g., antibiotic use) limited our ability to explore the potential impact of the maternal microbiota on that of the VPIs.

*Staphylococcus* is considered among the pioneer colonizers in the gut in VPIs, declining in abundance with advancing age and gut maturation (27, 59). The finding that they had the highest abundance in VPIs on BC may reflect a delayed GM development in these infants, potentially induced by a slightly higher intake of intact proteins, including immunoglobulins. Notably, the relative abundance of *Staphylococcus* was negatively correlated with body weight gain, consistent with previous results from another group (47). However, a recent meta-analysis could not confirm an association between *Staphylococcus* abundance and body weight gain (60), and the impact of these findings in BC-fortified VPIs remains to be verified.

Fecal pH values reflect changes in the gut environment, from the diet, the GM and/or derived metabolites (61). Lower fecal pH is associated with higher colonization resistance against pathogenic bacteria (62), higher abundance of bifidobacteria (63), and bioavailability of minerals, especially calcium (64). In our study, higher fecal pH in BC-fortified VPIs was correlated with higher relative abundance of *Staphylococcus* and lower *Bifidobacterium* abundance. The latter is in accord with previous reports (63, 65), possibly reflecting that *Bifidobacterium* are efficient acetate producers. If certain intact proteins from BC, such as casein and immunoglobulins, “escape” digestion in the small intestine, these proteins will be fermented in the colon releasing alkaline metabolites, such as ammonia, potentially increasing pH (66).

The present study represents the hitherto largest analysis of the effect of a dietary fortification intervention on the GM of VPIs. The samples included were from a well-designed and well-conducted clinical trial with detailed clinical conditions with detailed data collected for confounder adjustment in statistical analysis to secure a credible assessment of the effect of fortifiers. Our data are restricted to VPIs in a high-income setting with access to DHM. Lacking information about the digestibility of fortifiers complicates our interpretation of the GM changes observed. Another limitation is the amplicon sequencing approach based on the V3 hypervariable region. As for all common amplicon sequencing approaches using short-read technology, accurate species identification is challenging.

Collectively, the present study shows that the choice of fortifier, BC or CF, does influence GM composition in VPIs early in life, but the effect is modest. In contrast, birth mode more strongly influences GM shortly after birth, but with diminishing influence over time. Our finding is in line with other reports and corroborates the notion that the choice of fortifier has limited effects on GM development in the first month of life.

## Data Availability

The 16S rRNA gene amplicon sequencing data that support the findings of this study is openly available in the Sequence Read Archive at NCBI with the accession number PRJNA1090537 in an anonymized form. The clinical data containing personally identifiable information of individual infants participating in the cohort generated during the current study are not publicly available. This is due to the Danish Data Protection Act and European Regulation 2016/679 of the European Parliament and of the Council (GDPR), which prohibit distribution even in the pseudo-anonymized form to protect the privacy of the participants and their families. However, access to the data can be made through a joint research collaboration by contacting Gitte Zachariassen (Gitte.Zachariassen@rsyd.dk).

## SUPPLEMENTAL MATERIAL

Supplemental material msystems.00916-24-s0001.docx Fig. S1 to S5; Tables S1 to S4.

ASM does not own the copyrights to Supplemental Material that may be linked to, or accessed through, an article. The authors have granted ASM a non-exclusive, world-wide license to publish the Supplemental Material files. Please contact the corresponding author directly for reuse.
